# RNA Interference of 1-Aminocyclopropane-1-carboxylic Acid Oxidase (*ACO*1 and *ACO*2) Genes Expression Prolongs the Shelf Life of Eksotika (*Carica papaya* L.) Papaya Fruit

**DOI:** 10.3390/molecules19068350

**Published:** 2014-06-19

**Authors:** Rogayah Sekeli, Janna Ong Abdullah, Parameswari Namasivayam, Pauziah Muda, Umi Kalsom Abu Bakar, Wee Chien Yeong, Vilasini Pillai

**Affiliations:** 1 Malaysian Agricultural Research and Development Institute (MARDI), P.O. Box 12301, Kuala Lumpur 50774, Malaysia; E-Mails: lynn@mardi.gov.my (R.S.); pauziah@mardi.gov.my (P.M.); uab@mardi.gov.my (U.K.A.B.); cywee@mardi.gov.my (W.C.Y.); 2 Department of Microbiology, Faculty of Biotechnology & Biomolecular Sciences, Universiti Putra Malaysia, UPM Serdang 43400, Selangor Darul Ehsan, Malaysia; 3 Department of Cell and Molecular Biology, Faculty of Biotechnology & Biomolecular Sciences, Universiti Putra Malaysia, UPM Serdang 43400, Selangor Darul Ehsan, Malaysia; E-Mail: parameswari@upm.edu.my; 4 Ministryof Science, Technology and Innovation (MOSTI), Putrajaya 62574, Federal Territory, Malaysia; E-Mail: vilasini@mosti.gov.my

**Keywords:** Eksotika 1 papaya, RNA interference, ACC oxidase, *Agrobacterium* mediated transformation

## Abstract

The purpose of this study was to evaluate the effectiveness of using RNA interference in down regulating the expression of 1-aminocyclopropane-1-carboxylic acid oxidase gene in Eksotika papaya. One-month old embryogenic calli were separately transformed with *Agrobacterium* strain LBA 4404 harbouring the three different RNAi pOpOff2 constructs bearing the 1-aminocyclopropane-1-carboxylic acid oxidase gene. A total of 176 putative transformed lines were produced from 15,000 calli transformed, selected, then regenerated on medium supplemented with kanamycin. Integration and expression of the targeted gene in putatively transformed lines were verified by PCR and real-time RT-PCR. Confined field evaluation of a total of 31 putative transgenic lines planted showed a knockdown expression of the targeted *ACO*1 and *ACO*2 genes in 13 lines, which required more than 8 days to achieve the full yellow colour (Index 6). Fruits harvested from lines pRNAiACO2 L2-9 and pRNAiACO1 L2 exhibited about 20 and 14 days extended post-harvest shelf life to reach Index 6, respectively. The total soluble solids contents of the fruits ranged from 11 to 14° Brix, a range similar to fruits from non-transformed, wild type seed-derived plants.

## 1. Introduction

RNA interference (RNAi) is a recent technique that is used to knock-out or knock-down targeted gene expression. It presents a simple and powerful technique to understand plant gene expression and plant gene function [1]. RNAi involves gene silencing, whereby mRNA degradation is induced by short double-stranded RNA molecules, known as small interfering RNAs (siRNAs). siRNAs are complimentary in sequence to a known mRNA and are present in cells specifically to destroy that particular mRNA. The first RNAi-related phenomenon was observed in petunias, when Napoli *et al.* [2] discovered the introduction of a pigment-producing gene under the control of the cauliflower mosaic virus 35S promoter suppressed expression of both the introduced gene and its homologous endogenous gene. This phenomenon was called “co-suppression”. RNAi technology has been used to manipulate crop plants for improved agricultural traits such as pest and disease resistance, enhanced nutritional value, increased shelf-life and much more.

Globally, RNAi has been used successfully to down-regulate the biosynthesis of flavonoids in tomato by manipulating the production of the key enzyme, chalcone synthase [3]. Research has also been carried out in tomato to suppress the ethylene receptor, LeETR4, using the RNAi technique, which resulted in an early ripening tomato but with the fruit size, flavour and yield unchanged [4]. RNAi was considered good for this purpose as it is highly gene-specific, and can down-regulate or completely knock out a gene, if required. One specific gene or several genes can be silenced if the target sequence is chosen precisely. RNAi also has the ability to target multiple gene family members.

In Malaysia, RNAi technology has yet to be applied successfully to generate transgenic plants for local crop improvement. In this study, RNAi technology was applied to introduce the ripening- related genes, ACC oxidase 1 (*ACO*1) and ACC oxidase 2 (*ACO*2), into embryogenic calli of Eksotika papaya in order to produce delayed ripening papaya fruits. ACC oxidases are key enzymes in ethylene biosynthesis [5]. Among the major papaya varieties produced in Malaysia for export is Eksotika papaya, which resulted from a cross between Subang 6 and the Hawaian Sunrise Solo. It was released by the Malaysian Agricultural and Research Development Institute (MARDI) in 1987 [6]. The fruit has an orange-red flesh with a pleasant aroma, a high sugar content (12–14° Brix) and a soft texture. Despite having many attractive features, Eksotika papaya’s key deficiency is its fast ripening attribute. Considering the poor shelf-life of papaya fruit, this issue needs to be solved to prevent economic losses to the growers and the country as a whole. Hence, manipulation of the gene(s) involved in papaya fruit ripening was targeted in order to improve the shelf-life of future harvests of Eksotika papaya to more than 4 keeping days as exhibited by a normal Eksotika papaya.

## 2. Results and Discussion

### 2.1. Development of RNAi Constructs Using Gateway Technology

**Figure 1:**
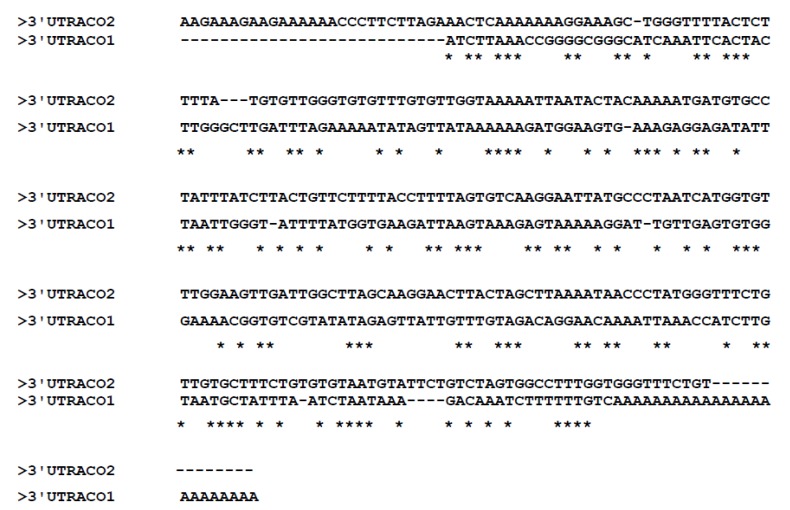
Sequence alignment of 3′UTR region of *ACO*1 and *ACO*2 isolated from Malaysian Eksotika papaya [12]. The DNA sequences of both *ACO* show high diversity. Asterisks (*) indicate the similarity of both *ACO* sequences.

The inducible silencing vector, pOpOff2 RNAi, from The Commonwealth Scientific and Industrial Research Organisation (CSIRO) was used for all Eksotika papaya constructs harbouring the two ACC oxidase genes, *ACO*1 and *ACO*2, isolated from Eksotika 1 papaya. *ACO*1 and *ACO*2 have high sequence similarity in their open reading frames while differences are located in the 5′ and 3′ untranslated regions (UTR) as shown in Figure 1 [7]. The target site for the RNAi constructs development was aimed at the 3′UTR regions of *ACO*1 and *ACO*2, respectively. Many studies reported the use of the 3′UTR region of the gene of interest to generate dsRNA [8,9,10] because it is more specific and effective, and is believed to affect mRNA stability and translational regulation [11]. In Eksotika papaya, the sequences of the 3′UTR region of *ACO*1 and *ACO*2 showed high diversity, and therefore, this region is specific for each gene.

**Table 1:** PCR analyses of RNAi putative transgenic papaya plants.

Construct	Total Number of Callus Transformed	Number of Putative Transgenic Lines Obtained	PCR Positive for *npt*II	PCR Positive for *gus*A and PDK Intron	PCR Positive for Gene of Interest	Number of Regenerated Putative Transgenic Plants
pRNAiACO1	5,000	80	68	68	68	68
pRNAiACO2	5,000	30	26	26	26	26
pRNAiCACO	5,000	66	54	54	54	54
Total	15,000	1.17% (of total calli transformed)	0.99% (of total calli transformed)	0.99% (of total calli transformed)	0.99% (of total calli transformed)	100% (of total positive putative transgenic lines obtained)

### 2.2. Agrobacterium Transformation and PCR Analysis

A total of 176 putative transformants were obtained from 15,000 transformed embryogenic calli after 4 months selection on kanamycin medium. The presence of the transgenes in putative transformed lines were verified by PCR analysis and the results showed that 148 out of 176 lines tested positives for *npt*II, *gus*A, PDK intron, *ACO*1 and *ACO*2 genes (data not shown). All the genes were successfully co-transformed (100%) into the plant genome. Overall, the PCR transformation efficiency was 0.99%, calculated based on the total number of PCR positives for the entire gene types tested per total number of calli transformed (Table 1). All the putative transformed papaya plants were successfully regenerated into plantlets with a high survival rate of 92% after transferred into soil (ground).

### 2.3. Gene Expression Analysis

**Figure 2:**
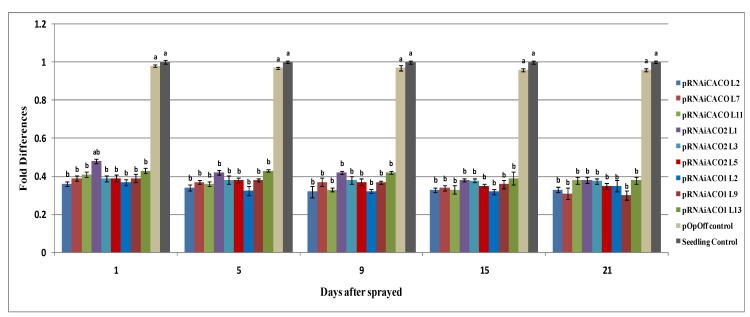
Gene expression analysis of putative transgenic plants harbouring pRNAiACO1, pRNAiACO2 and pRNAiCACO after treatment with dexamethasone. pOp: plant transformed with RNAi pOpOff vector, and Non- transformed control: Seedling-derivednon-transformedplant. Duncan multiple range test was used to compare the mean values among the test at 95% probability. Means with the same letters are not significantly different at *p* < 0.05. *n* = 3 replicates.

The ability of the inducible pOp6 promoter to influence the *ACO*1 and *ACO*2 gene expression was tested in three different putative transgenic lines grown in soil in polybag for each RNAi construct by watering with 2 µM dexamethasone. There are many approaches reported which can be used to apply dexamethasone to the plant at different developmental stages such as adding directly to the culture medium, watering, spraying, and wiping of the leaf [13,14,15]. Dexamethasone can also be applied during tissue culture by supplementing it in the media or in the field *via* watering or spraying. Application of dexamethasone during tissue culturing process might be more effective for induced expression in studying gene function compared to adding to the mature plant in soil because the *in vitro* plants have more excess to dexamethasone in the media from initial germination until rooting [16]. It was reported that watering was better as it resulted in activation of pOp6/LhGR throughout the plant compared to painting the leaf where it resulted in induction throughout the leaf only [13]. Therefore, the approached of watering the plants was selected in this study. Two µM of dexamethasone was chosen based on the concentration reported by [17] who reported that pOp6/LhGR was maximally induced at this concentration. In the current study, gene expression analyses were carried out after 1, 5, 9, 15 and 21 days post-induction with dexamethasone (Figure 2).

Many studies reported the reduction of targeted gene expression could be achieved within 24 h in many organisms by using double stranded RNA [18,19,20]. In our study, we observed that expressions of the targeted *ACO*1 and *ACO*2 genes were down-regulated 2.5-fold after 1 day of treatment and the levels were similar from day-5 to day-21 post-treatment. A single dose of dexamethasone application was found to be sufficient to activate the pOp6 promoter. The expression level in the non-induced putative transgenic RNAi plants was not significantly different (*p* < 0.05) from the non-transformed plants. These results indicated that there was no leakage of transgenes expression, because the pOp6 promoter used is very specific to the inducer dexamethasone. This proved that this type of promoter is suitable to be applied for functional gene studies especially for those involved in production of final products that affect plant development. The results obtained showed that the expression of the targeted genes in the transformed papaya could be knocked down by using RNAi approach. However, it is critical to note that the concentration of dexamethasone used in this study should not be considered as the optimal concentration for induction in mature field grown papaya plants. If the study is to be carried out on field grown papaya plants, further optimization of the amount of dexamethasone used need to be evaluated for maximal knock down.

### 2.4. Field Evaluation and Fruit Analysis

In general, the growth performance of the RNAi transgenic plants was quite similar in terms of plant height, stem girth and mean internode length to the seed-derived non-transformed plants. The plant height, stem girth and mean internode length were observed to be non-significantly different at 189.0 ±23.5 cm, 11.8 ± 2.5 cm and 3.5 ± 0.6 in RNAi transgenic plants, and 210 ± 20.2 cm, 12.4 ± 3.1 cm and 3.5 ± 0.2 cm in wild type seed-derived non-transformed plants, respectively.

Shelf life analyses of the fruits were carried out *via* direct application of dexamethasone on the harvested fruits harvested of Index 2, and not on the RNAi transgenic plants in the field. This was done to avoid dexamethasone from activating the promoter to indirectly silence ethylene gene expression, which then affects the plant’s growth and development [21]. Although some researchers [13,17] reported that the watering method was more efficient to induce the pOp6 promoter, the spray method applied on Index 2 harvested fruit was used in this study because dexamethasone is a controlled drug and there is a need to comply with the Malaysian Biosafety Act 2007.

Fruits from 28 transgenic lines (30 lines were analysed) showed delayed ripening in comparison to the wild type seed-derived non-transformed fruits. Earlier findings by Salleh *et al.* [22] showed that a wild type seed-derived non-transformed Eksotika fruit required 4 days to achieve full colour Index (Index 6). Overall, the days to achieve the full yellow colour Index (Index 6) by the transformed fruits in this study ranged from 5 to 20 days. However, 13 transgenic lines had fruits that required more than 8 days to achieve full ripening. Fruits harbouring construct developed based on the conserved region of *ACO*1 and *ACO*2 (designated as pRNAiCACO) had shorter shelf life span (maximum 11 days) compared to those harbouring constructs developed using the 3′UTR region of *ACO*1 (designated as pRNAiACO1) and *ACO*2 (designated as pRNAiACO2) genes (maximum 14 and 20 days).

**Figure 3:**
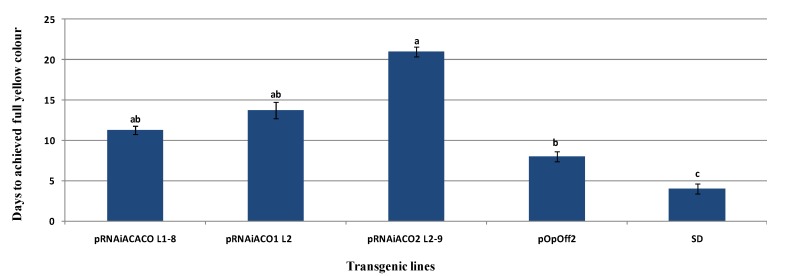
Shelf life analysis of RNAi Transgenic Papaya Plants. Lines pRNAiCACOL1-8, pRNAiACO1 L2 and pRNAiACO2 L2-9 are the transgenic lines. pOpOff2 is the vector transformed fruit, and SD: Seedling-derived non-transformed fruits. Duncan multiple range test was used to compare the mean values among the test at 95% probability. Means with the same letters are not significantly different at *p* < 0.05.

For fruit transformed with pRNAiACO1, four transgenic lines (pRNAiACO1-Line1, -Line 2, -Line 3, and -Line 36) required more than 10 days to achieve Index 6, with the longest being 14 days. However, the fruits harbouring pRNAiACO2 had an even longer shelf-life of 22 days after harvest to reach Index 6 (Figure 3). The results obtained were encouraging as they suggested that *ACO*1 and *ACO*2 manipulation have potentials to prolong the shelf life of the fruits, especially *ACO*2. Previously, Sew *et al.* [7] found that *ACO*2 in Malaysian Eksotika papaya was upregulated during fruit ripening and highlighted that *ACO*1 was more involved in plant development. The results obtained in this study further substantiated Sew *et al.* [7] findings that *ACO*2 does play a more pivotal role in the Eksotika papaya fruit ripening process compared to *ACO*1, and the main targeted region was the 3′UTR *ACO*2. Targeting the UTR region to generate dsRNA had been reported to be more specific for gene function [9]. The study by Mazumder *et al.* [11] showed that the UTR region plays a role in mRNA stability and translational regulation. The shelf-life analysis data proved that the RNAi constructs successfully reduced the *ACO* gene expression with different levels of reduction. Variable patterns of shelf life’s span amongst the different transgenic line that carry the same transgene was observed in this study. This might be due to different insertional chromosal locations of the transgene, which affect its expression. Such variability in transgene expression level due to different integration positions is a common phenomenon in plant transformation [23,24]. Other possible factors that might contribute to this different expression include the copy number and transgene construct fidelity [24,25]. A comparison of the total soluble solid between RNAi transgenic and wild type seed-derived control fruit showed similar profile of 11–14° Brix. These results imply that the transgenes did not affect the TSS content, and this is very important in gene manipulation of papaya where it should not alter the original plant nutrients.

## 3. Experimental Section

### 3.1. Development of RNA Interference (RNAi) Constructs

**Figure 4:**
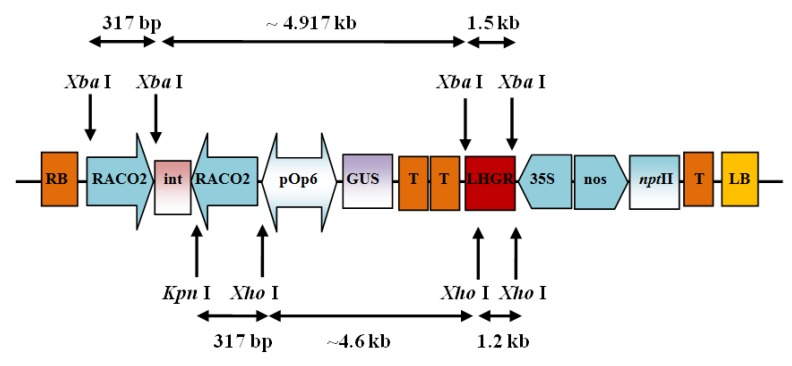
Construct map of pRNAiACO2 (RACO2) construct with *ACO2* gene. The sequence base pair length for pRNAiACO2 (317bp) construct is as indicated. RB: T-DNA right border, LB: T-DNA left border, int: pyruvate orthophosphate dikinase intron, pOp6: pOp6 promoter, 35S: cauliflower mosaic virus 35S RNA promoter, nptII: kanamycin resistance gene, GUS: β-glucoronidase gene, nos: nopaline synthase promoter, T: terminator. Letters on the top and bottom rows indicate the restriction enzyme sites for *Xba*I, *Kpn*I and *Xho*1.

The RNAi constructs developed were designed to knockdown expression of the ACC Oxidase 1 (*ACO*1) and ACC Oxidase 2 (*ACO*2) genes in transgenic Eksotika papaya. In order to knockdown the expression of *ACO*1, the target RNAi sequence was amplified from the 3′UTR of *ACO*1 mRNA. This gave rise to PCR fragment of 350 bp and the construct harbouring the targeted gene was designated, pRNAiACO1. Likewise, to down-regulate expression of the *ACO*2 gene, amplification of the target sequence was directed from the 3′UTR of *ACO*2 mRNA resulting in a PCR fragment of 317 bp. This construct was designated, pRNAiACO2 (Figure 4). To knock-down both the *ACO*1 and *ACO*2 genes, a conserved region of both genes was amplified to use as a target sequence with a target size of 341 bp. This construct was designated, pRNAiCACO (Table 2). Each PCR reaction consisted of 1X PCR buffer, 25 mM MgCl_2_, 2 mM dNTPs, 10 μM each of the sense and antisense primers, 3U Taq DNA polymerase, 300 ng of plasmid DNA, and the final volume was adjusted to 20 μL with sterile distilled water. The PCR conditions were a pre-denaturation step at 94 °C, for 1 min, followed by 34 cycles of amplication at 94 °C for 1 min, 60 °C for 45 s and 72 °C for 1 min. A final extension step of 10 min at 72 °C completed the process.

The PCR-amplified products were cloned respectively into an intermediate vector, PCR8/GW/TOPO, before sub-cloning into the destination RNAi vector, pOpOff2, using the Gateway (Invitrogen, California, USA) directed recombination system. The inducible RNAi vector (pOpOff2) used in this study used dexamethasone as an inducer to activate gene expression. The PCR products from the *ACO* gene target regions were verified by restriction enzyme digestion using *Xba* I, PCR, and followed by DNA sequencing. All gene constructs were transformed into *Agrobacterium* *tumefaciens* strain LBA 4404 by electroporation. Briefly, transformation was carried out using 3 μg of each respective plasmid construct to *Agrobacterium* LBA 4404 competent cell, mixed by pipetting, then transferred directly into an electroporation cuvette. The cells were then pulsed immediately using a Bio-Rad electroporator (Bio-Rad, California, USA) and used for the transformation of embryogenic calli of Eksotika papaya.

**Table 2:** Sequence of the primers used for developing RNAi constructs.

Primer	Orientation	Sequence (5'-3')	Length (bp)	Amplicon size (bp)
pRNAiACO1	Sense	cttctttctacaaccccagc	20	350
	Antisense	gacaccgttttcccacact	19	
pRNAiACO2	Sense	tctactgtaactcctggtgc	20	317
	Antisense	caccaaaggccactagacag	20	
pRNAiCACO	Sense	atgaaggagtttgcagtggg	20	341
	Antisense	ccgttagtaatcagctcaag	20	

### 3.2. Papaya Transformation and Regeneration

One-month-old embryogenic calli were induced from immature embryos of Eksotika papaya and transformed with RNAi constructs (pRNAiACO1, pRNAiACO2 and pRNAiCACO) using an established method for *Agrobacterium-*mediated transformation of Eksotika papaya [26]. The calli were incubated at 28 °C with *Agrobacterium* of OD_600_ = 0.5 for 40 min and the transformed calli were selected on half-strength Murashige and Skoog (MS) basal salts medium [27] supplemented with kanamycin. Selection was carried out for a total of four months. The selection was carried out with 75 mg/L kanamycin for 1 month, followed by 150 mg/L kanamycin for the remaining 3 months. The kanamycin resistant calli that proliferated on the selection media were then transferred to De Fossard [28] maturation medium (De Fossard medium without any plant growth regulator) for 1 month. Proliferating calli with green shoots were then cultured on De Fossard regeneration medium supplemented with 0.89 µM 6-benzyladenine (BA), 1.1 µM α-naphthaleneacetic acid (NAA) and 150 mL coconut water for shoot regeneration. Putative transgenic shoots were then cultured on half-strength liquid MS medium supplemented with vermiculite for rooting, and the rooted plantlets consisting of at least four lateral branches and abundant root hairs were transferred to growing medium consisting of a 1:1:1 mix of soil: sand: vermiculite for acclimatisation.

### 3.3. Analysis of RNAi Transgenic Plants

Approximately, 100 mg of leaf tissue from each putative transformed or non-transformed plant were used to isolate DNA using the Qiagen Miniprep kit (Qiagen, Hilden, Germany). Fifty ng of each extracted genomic DNA samples was used for PCR analysis. PCR verification was done for the *npt*II, *gus*A, pyruvate dehydrogenase kinase (PDK) intron and specific *ACO*1 and *ACO*2 genes. The list of primers used is as shown in Table 3 and the thermal cycling conditions were as follows: 2 min at 94 °C; 35 cycles of 30 s at 94 °C, 45 s at 62 °C and 1 min 30 s at 72 °C; and finally at 72 °C for 10 min. Twenty microliters of each PCR amplified products were electrophoresed on 1.0% (w/v) agarose gel.

**Table 3:** List of primers used for analysis of RNAi transgenic papaya plants.

Name	Sequence (5'-3')	Length (bp)	Amplicon Size (bp)
*npt*IIF	ccttatccgcaacttctttacc	22	610
*npt*IIR	caccatgatattcggcaagcag	22	
*gus*AF	catggtacgtcctgtagaaacc	22	1711
*gus*AR	gaagatccctttcttgttaccg	22	
PDK IntronF	ttcccaactgtaatcaatcc	20	622
PDK IntronR	tgacaagtgatgtgtaagac	20	
pRNAiACO1F	cttctttctacaaccccagc	20	350
pRNAiACO1R	gacaccgttttcccacact	19	
pRNAiACO2F	tctactgtaactcctggtgc	20	317
pRNAiACO2R	caccaaaggccactagacag	20	
pRNAiCACOF	atgaaggagtttgcagtggg	20	341
pRNAiCACOR	ccgttagtaatcagctcaag	20	

### 3.4. Determination of the Effect of Dexamethasone on RNAi Transgenic Papaya

A preliminary investigation on the ability of the inducible pOp6 promoter to influence the *ACO*1 and *ACO2* gene expression was performed on three positive transformant papaya plants for each RNAi construct. The RNAi papaya plants for each RNAi construct and non-transformed seedlings (controls) were watered three times on 1 day with 200 mL freshly prepared 2 µM dexamethasone. Analyses of gene expression were carried out after 1, 5, 9, 15 and 21 days post induction. Three putative transgenic lines for each RNAi construct, and RNA samples from three plants in each line were used as replicates for the analysis. Total RNA was isolated from approximately 100 mg of young leaves tissue of each RNAi transgenic and control papaya plant using the RNeasy Minit kit (Qiagen, Hilden, Germany). One µg of total RNA was converted into first strand cDNAs using QuantiTect Reverse Transcription kit (Qiagen, Hilden, Germany) and used as template for gene expression studies by real-time RT-PCR. Real-time RT-PCR analysis was carried out using an ABI PRISM 7700 system (Applied Biosystems, Foster, CA, USA). Each real-time RT-PCR reaction contained 100 ng of first strand cDNA, 1 X SYBR green master mix, 200 nM each sense and antisense primers and the final reaction volume was adjusted to 20 µL using sterile distilled water. The PCR cycling conditions were a pre-denaturation step at 94 °C for 2 min, followed by 40 cycles repetition of the following steps: 94 °C denaturation for 15 s, 55 °C annealing for 30 s, 72 °C extension for 30 s and a final step at 95 °C for 1 min. Quantification of the relative levels of gene expression was performed in which expression of the target in each plant sample was calculated relative to a housekeeping gene. Three housekeeping genes sequence encoding actin, 18S ribosomal RNA and a 40S ribosomal protein were used. Each DNA template was analysed three times and the experiment was repeated twice. The average of two biological replicates was used for correlation analysis.

### 3.5. Field Evaluation and Fruit Analysis

Regenerated transgenic papaya R_0_ plants were hardened and acclimatized in contained environment, transgenic glasshouse before planting under nethouse condition. Both transgenic and non-transgenic plants were planted in completely randomized design. The planting distance of the plants was 2.2 m between rows and 2 m within rows. Thirty-one independent RNAi transgenic plants were grown in soil under insect-proof netting to assess their morphologicly and the shelf-life characteristics of the transgenic papaya fruit. Morphological analysis of R_0_ transgenic plants was carried out based on quantitative characters such as plant height, stem girth, height of first flower, height of first fruits and mean internode lengths at 1 month intervals. Fruit shelf-life analyses were examined daily by scoring the colour indices of the papaya fruits harvested at Index 2 until the fruits achieved Index 6 (full yellow). Fruit harvesting process was carried out at 8 in the morning and fruits were sprayed with 2 µM dexamethasone immediately after harvest to activate *ACO* gene expression. The Shelf-life of the fruits was examined 24 h after induction. Total soluble solids (TSS) concentrations were measured for each fruit that achieved the full ripening stage (Index 6) using a Digital Refractometer (Model PR-1; ATAGO CO, Tokyo, Japan).

## 4. Conclusions

This study showed that manipulation of two *ACO* gene(s) in Eksotika papaya by RNA interference increased the post-harvest shelf-life of transgenic fruits. Several promising transformant lines bore fruits having an approximate three to four times longer shelf life than the normal post-harvest life. Such RNAi manipulation is specific, and the total soluble solid of fruit was comparable to the non-transformed seedling-derived fruits, indicating that transformation had not compromised fruit quality.
